# Draft Genome Sequences of Eight Mycobacterium montefiorense Strains Isolated from Salamanders in Captivity

**DOI:** 10.1128/mra.00702-22

**Published:** 2022-10-31

**Authors:** Takeshi Komine, Hyogo Ihara, Hanako Fukano, Yoshihiko Hoshino, Osamu Kurata, Shinpei Wada

**Affiliations:** a Laboratory of Aquatic Medicine, School of Veterinary Medicine, Nippon Veterinary and Life Science University, Musashino, Tokyo, Japan; b Department of Mycobacteriology, Leprosy Research Center, National Institute of Infectious Diseases, Higashi-Murayama, Tokyo, Japan; Montana State University

## Abstract

Mycobacterium montefiorense is a nontuberculous mycobacterium that causes infections in fish and salamanders. Here, we report annotated draft genome sequences of eight strains that were isolated in 2014 and 2018 from salamanders reared in an aquarium in Japan.

## ANNOUNCEMENT

Mycobacterium montefiorense is a nontuberculous mycobacterium (NTM) that causes mycobacteriosis in fish and salamanders (1–3). Mycobacterium montefiorense has also been isolated from soil and pond water (4). We sequenced the genomes of eight M. montefiorense strains that were isolated in 2014 and 2018 from salamanders reared in Niigata City Aquarium (Niigata, Japan).

Several dead salamanders in the aquarium were collected (Table 1) and routinely dissected. The liver tissues were sampled, homogenized, and decontaminated with 1 mL of *N*-acetyl-l-cysteine-sodium citrate-NaOH for no more than 15 min. After neutralization with 6 mL of phosphate buffer (pH 6.8), the samples were centrifuged; the pellets obtained were then inoculated on Middlebrook 7H10 agar supplemented with 10% BBL Middlebrook oleic acid-albumin-dextrose-catalase (OADC) enrichment (Becton, Dickinson and Company, USA) and on 2% Ogawa egg slants (Kyokuto Pharmaceutical Industrial Co., Ltd., Japan). Isolates obtained were identified as M. montefiorense based on the Runyon classification system (5) and phylogenetic analysis of the 401-bp 65-kDa heat shock protein gene (*hsp65*) with the Tb11/Tb12 primer set (6).

**TABLE 1:** Strain information and assembly statistics

Strain	Isolate source	Year of isolation	No. of raw reads	Genome size (bp)	No. of contigs	*N*_50_ (bp)	Coverage (×)	Total no. of CDSs^*a*^	G+C content (%)	SRA accession no.	Contig accession no.
NJB14191	Hakuba salamander (Hynobius hidamontanus)	2014	3,646,896	5,744,673	123	171,591	85	5,348	65.1	DRR357474	BQYA00000000
NJB14192	Hakuba salamander (H. hidamontanus)	2014	1,841,220	5,776,754	234	157,068	43	5,366	65.1	DRR357475	BQYB00000000
NJB14194	Hakuba salamander (H. hidamontanus)	2014	1,946,204	5,753,077	155	169,431	46	5,382	65.1	DRR357476	BQYC00000000
NJB14195	Hakuba salamander (H. hidamontanus)	2014	2,424,346	5,749,641	116	201,183	58	5,381	65.1	DRR357477	BQYD00000000
NJB14197	Hakuba salamander (H. hidamontanus)	2014	3,047,344	5,745,062	108	201,635	72	5,364	65.1	DRR357478	BQYE00000000
NJB18182	Tohoku hynobiid salamander (Hynobius lichenatus)	2018	2,308,180	5,764,439	210	136,848	52	5,352	65.1	DRR357479	BQYF00000000
NJB18183	Tohoku hynobiid salamander (H. lichenatus)	2018	2,811,918	5,749,386	127	207,711	64	5,376	65.1	DRR357480	BQYG00000000
NJB18185	Tohoku hynobiid salamander (H. lichenatus)	2018	4,174,608	5,739,217	88	240,008	99	5,381	65.1	DRR357481	BQYH00000000
ATCC BAA-256	Green moray (Gymnothorax funebris)	NC^*b*^	8,308,120	5,226,877	735	16,108	396	4,149	65.2	DRR361296	BSAJ00000000

a CDSs, coding sequences.

b NC, not clear.

Frozen stocks (−80°C in 20% glycerol) of M. montefiorense strains were streaked on 2% Ogawa slants, and single colonies were grown at 25°C for approximately 4 weeks. The collected colonies were boiled at 95°C for 15 min, frozen at −20°C overnight, and disrupted twice (4,500 rpm for 1 min) with approximately 0.5-mm-diameter zirconia/silica beads (BioSpec Products, Inc., USA) using a Micro Smash MS-100 disrupter (Tomy Digital Biology Co., Ltd., Japan). Genomic DNA was extracted using the NucleoSpin Plant II kit (Macherey-Nagel GmbH & Co. KG, Germany) in accordance with the manufacturer’s instructions.

Sequencing libraries were prepared using the Nextera XT DNA library preparation kit (Illumina, USA) and sequenced on the Illumina HiSeq X platform (2 × 150 bp). The quality of the raw reads was assessed with FastQC v0.11.9 (7). The sequence reads were trimmed for quality using fastp v0.20.1 (8) and assembled *de novo* using Platanus_B v1.1.0 (9). We also assembled the genome of the type strain (M. montefiorense ATCC BAA-256) using the raw data (accession number DRR361296) from the National Center for Biotechnology Information (NCBI) GenBank database using the same method as described above. Automated annotation was conducted with the DNA Data Bank of Japan (DDBJ) Fast Annotation and Submission Tool (DFAST) (https://dfast.ddbj.nig.ac.jp). The annotated assemblies for eight strains and the type strain were deposited in the DDBJ. All genomic statistics are given in Table 1. Default parameters were used for all software unless otherwise noted.

**FIG 1:**
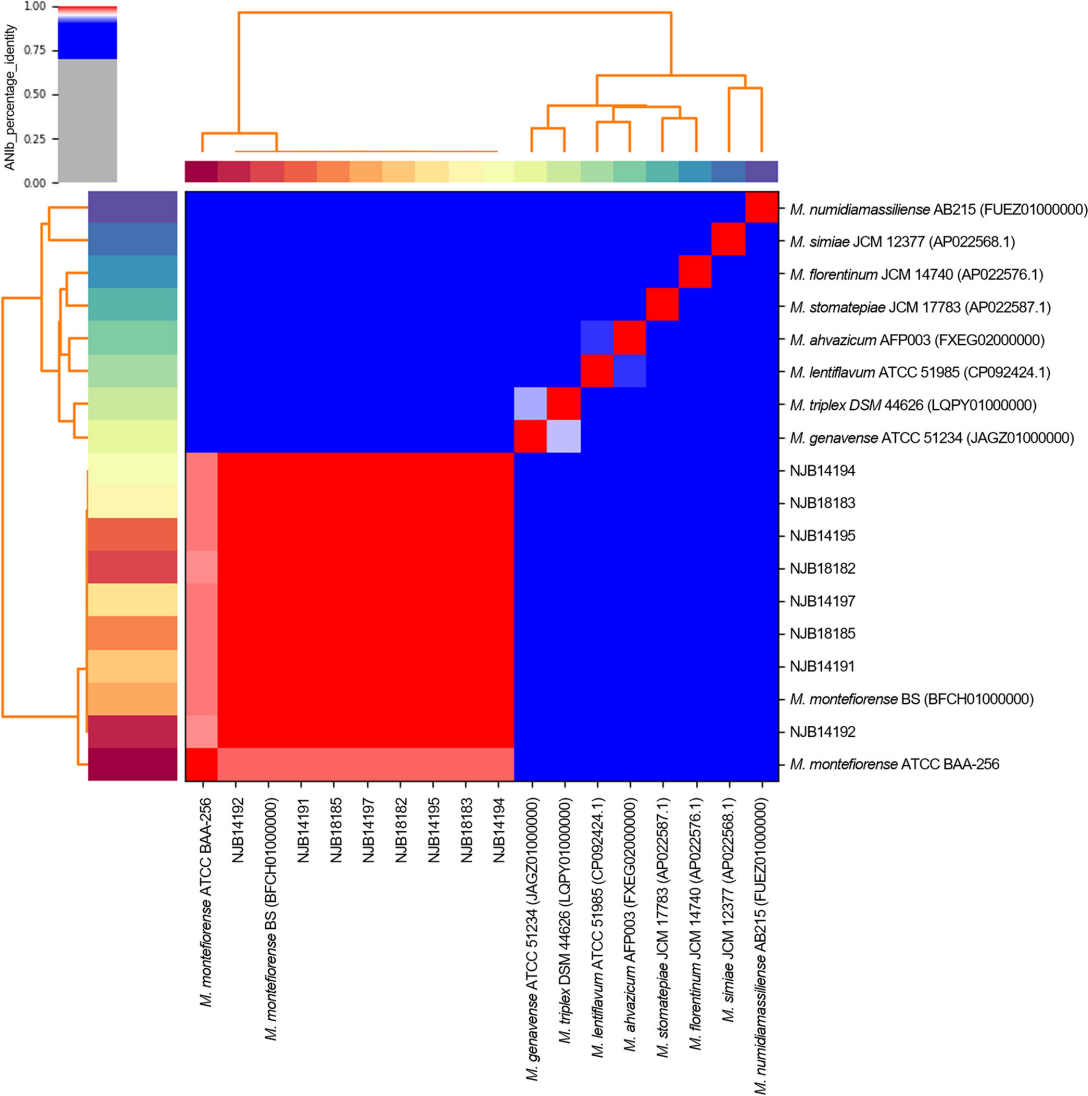
Heatmap of ANI values for 18 Mycobacterium strains. The heatmap was generated using pyani v0.2.11 and a BLAST-based approach (ANIb).

Average nucleotide identity (ANI) analysis was conducted to determine relationships among Mycobacterium species. ANI values were determined for the whole-genome sequences using pyani v0.2.11 and a BLAST-based approach (ANIb) (10). The ANI heatmap is shown in Fig. 1, and the ANI values for comparisons between the eight strains and M. montefiorense ATCC BAA-256 were >97.2%.

We report the draft genome sequences of eight M. montefiorense strains that were isolated from salamanders in captivity. These sequences will improve our understanding of the pathogenicity and evolution of this mycobacterial species.

### Data availability.

The genome sequencing and assembly projects have been deposited in the DDBJ under BioProject accession number PRJDB13312. See Table 1 for the DDBJ Sequence Read Archive (DRA) and DDBJ accession numbers.
